# Regional Intestinal Drug Absorption: Biopharmaceutics and Drug Formulation

**DOI:** 10.3390/pharmaceutics13020272

**Published:** 2021-02-17

**Authors:** Arik Dahan, Isabel González-Álvarez

**Affiliations:** 1 Department of Clinical Pharmacology, School of Pharmacy, Faculty of Health Sciences, Ben-Gurion University of the Negev, Beer-Sheva 8410501, Israel; 2 Engineering, Pharmacokinetics and Pharmaceutical Technology Area, Miguel Hernandez University, 03550 Juan de Alicante, Spain

**Keywords:** biopharmaceutics, drug absorption, drug solubility/dissolution, intestinal permeability, oral drug delivery, regional/segmental-dependent permeability and absorption

## Abstract

The gastrointestinal tract (GIT) can be broadly divided into several regions: the stomach, the small intestine (which is subdivided to duodenum, jejunum, and ileum), and the colon. The conditions and environment in each of these segments, and even within the segment, are dependent on many factors, e.g., the surrounding pH, fluid composition, transporters expression, metabolic enzymes activity, tight junction resistance, different morphology along the GIT, variable intestinal mucosal cell differentiation, changes in drug concentration (in cases of carrier-mediated transport), thickness and types of mucus, and resident microflora. Each of these variables, alone or in combination with others, can fundamentally alter the solubility/dissolution, the intestinal permeability, and the overall absorption of various drugs. This is the underlying mechanistic basis of regional-dependent intestinal drug absorption, which has led to many attempts to deliver drugs to specific regions throughout the GIT, aiming to optimize drug absorption, bioavailability, pharmacokinetics, and/or pharmacodynamics. In this Editorial we provide an overview of the Special Issue "Regional Intestinal Drug Absorption: Biopharmaceutics and Drug Formulation". The objective of this Special Issue is to highlight the current progress and to provide an overview of the latest developments in the field of regional-dependent intestinal drug absorption and delivery, as well as pointing out the unmet needs of the field.

Oral administration is without a doubt the most preferred and convenient way of drug delivery. Oral drug products are easy to use and usually do not require hospitalization or the assistance of medical staff. Oral intake of drugs may also prevent both the local side effects and the risk of systemic infections associated with injections. However, orally taken drugs must enter the enterocytes and cross through the gastrointestinal tract (GIT) membrane in order to reach the systemic circulation and exert their pharmacological effect. The GIT membrane acts as a barrier against the absorption of xenobiotics, and while some drugs easily overcome this barrier, many other drugs fail to penetrate through this membrane and have to be administered parenterally. These drugs include antibodies, protein/peptides, hormones, and even small molecules. Orally swallowed drugs are very much influenced by the physiological/biochemical conditions throughout the GIT, which may dictate their absorption potential, resulting in significant diversions from the predicted/desired pharmacokinetics and pharmacodynamics.

The GIT can be broadly divided to several regions: the stomach, the small intestine (which is subdivided into the duodenum, jejunum, and ileum), and the colon. The conditions and environment in each of these segments, and even within the segment, are dependent on many factors, e.g., the surrounding pH [1,2,3,4,5,6], fluid composition [7,8,9], transporters expression [10,11,12], metabolic enzymes activity [13,14], tight junction resistance [15,16], different morphology along the GIT [17,18], variable intestinal mucosal cell differentiation [19,20], changes in drug concentration (in cases of carrier-mediated transport), thickness and types of mucus [21], and resident microflora [22,23,24]. Each of these variables, alone or in combination with others, can fundamentally alter the solubility/dissolution, the intestinal permeability, and the overall absorption of various drugs [25,26,27,28]. This is the underlying mechanistic basis of regional-dependent intestinal drug absorption, which has led to many attempts to deliver drugs to specific regions throughout the GIT, aiming to optimize drug absorption, bioavailability, pharmacokinetics and/or pharmacodynamics.

The objective of this Special Issue is to highlight the current progress and to provide an overview of the latest developments in the field of regional-dependent intestinal drug absorption and delivery, as well as pointing out the unmet needs of the field.

Nowadays, the “3R’s Principle”, that is, replacement, reduction, and refinement, has greatly influenced scientific research in oral drug development, and several preclinical models have been developed to predict intestinal absorption process, aiming to reduce or even replace human/animal experiments. These methods are thoroughly evaluated in this Special Issue. The differences in anatomical and physiological features along the GIT complicate absorption predictions. Rezende’s group demonstrated that the BAMPA (biomimetic artificial membrane permeability assay) over Franz cell apparatus showed acceptable log-linear correlation (R^2^ = 0.664) with fraction of dose absorbed in humans (F_a_%), as seen for P_app_ in Caco-2 cells (R^2^ = 0.805), and, thus, both methods are acceptable for BCS classification [29]. Caco-2 cell predictability has been widely studied, and several authors demonstrated the correlation between Caco-2 cells and structure–activity of the drug [30,31,32]. In this issue, Huong Ta et al. developed a QSAR model (quantitative structure–activity relationship) using a machine learning-based hierarchical support vector regression (HSVR) scheme [33]. This tool allowed for the development of a model to predict Papp in Caco-2 cells permeability for drugs that are transported across the intestinal membrane not only in passive diffusion but also in transporter-mediated active transport. The group of Bermejo and González-Álvarez demonstrated that in vitro permeability studies in Caco-2 can be used to compare formulation performance and even to explain bioequivalence failures associated with excipient effect on the intestinal membrane [34]. In this study, Ruiz-Picazo et al. showed that permeability differences in rat vs. Caco-2 cells of pravastatin formulations (a BCS class III compound) can explain the bioequivalence failure and the higher C_max_ due to excipient effects on the intestinal membrane in the nonequivalent formulation. In the same article, the authors demonstrated non-similar dissolution profiles with USP apparatus and the relevance of using 500 mL instead of 900 mL [34]. A great research effort is focused on the development of new dissolution systems, mono- and multi-compartmental dynamics models. The adequate in vitro model should be chosen depending on the BCS classification of the studied drug and its physicochemical properties. These in vitro systems combine dissolution and absorption processes and simulate pH or peristaltic changes in luminal conditions; therefore, a mechanistic, physiologically based biopharmaceutics modeling (PBBM) approach to assess the in vivo performance of orally administered drug products (IVIVC) should be used to model the obtained data. These models are more complicated and require adequate software but generate better correlations between in vitro and in vivo values, as demonstrated by Bermejo at al. in their study of different ibuprofen formulations [35]. Several absorption models can be tested with different software, such as Phoenix WinNonlin® [35] or the GastroPlus™ simulator, as was demonstrated by Dahan's group [36], studying the rather complicated intestinal permeability of the BCS class IV drug furosemide that shows high dependency on many biochemical/physiological variables. Another common piece of software in the population pharmacokinetic area is NONMEM, which can be used in modelling absorption due to the flexibility of using custom-made empirical, semi-mechanistic, or physiological-based absorption approaches. Transit compartment models, absorption with and without lag time, or passive/active absorption kinetics models can be applied. In this context, Ruiz-Garcia et al. modeled dacomitinib absorption differences in the presence/absence of a proton pump inhibitor by comparing several physiologically based absorption models [37]. 

The behavior of controlled release formulations and the difficulties in predicting the human situation are studied in this Special Issue. Lennernäs et al. thoroughly discuss the predictive ability of rat colon studies in relation to human data and conclude that improved predictability is needed for controlled release formulations, and the use of permeation enhancers to increase colonic permeability could have higher risks than potential rewards [38]. Langguth's group studied the relevance of dissolution and disintegration of controlled release (CR) dosage forms, obtaining good correlations between the two processes [39]. This work may have significant regulatory impact, as it opens the way to extend the dissolution-based waiver concept beyond immediate-release dosage forms. However, the authors conclude that the extrapolation of these results to the in vivo situation should be done with caution due to additional factors that should be considered, e.g., transporter saturation effects, interplay with food and gastric emptying effects, and different hydrodynamics or mechanical stresses; these factors may complicate the correlation between disintegration and bioavailability [39]. Dahan's group investigated the role of segmental-dependent intestinal absorption in controlled release (CR) drug product development. The studied drug was carvedilol due to its zwitterionic nature; thus, this compound changes solubility in different conditions throughout the GIT [40]. The solubility, permeability, and dissolution of the drug were investigated in silico, in vitro, and in vivo, focusing on location-dependent effects. The authors demonstrated that a CR product could modify the drug solubility behavior from class II to I; these results are highly relevant in the decision-making process regarding the development of new CR drug products. The increased interest in drug delivery to the last segments of the GIT has led to new insights in the design of colonic delivery devices; Christfort et al. studied cylindrical, triangular, and cubic microcontainers with amoxicillin and showed that shape and surface texture of microcontainers influence the ex vivo mucoadhesion (cubic microcontainers are more adhered to intestinal mucus than cylindrical microcontainers), and the absorption of amoxicillin was higher from cubic microcontainers than from cylindrical or triangular microcontainers [41].

This Special Issue also includes two highly relevant review articles: the group of K. Sandy Pang focus on intestinal and liver metabolism and drug–drug interactions [42], and Tongzhi et al. focus on regional gut stimulation, concluding that the region of the gut exposed to intraluminal stimuli is of major relevance to the secretion profile of gastrointestinal hormones and associated metabolic responses [43].
